# Classification of the Epileptic Seizure Onset Zone Based on Partial Annotation

**DOI:** 10.1007/s11571-022-09857-4

**Published:** 2022-08-18

**Authors:** Xuyang Zhao, Qibin Zhao, Toshihisa Tanaka, Jordi Solé-Casals, Guoxu Zhou, Takumi Mitsuhashi, Hidenori Sugano, Noboru Yoshida, Jianting Cao

**Affiliations:** 1 Department of Electrical Engineering and Computer Science, Tokyo University of Agriculture and Technology, Tokyo, Japan; 2 Tensor Learning Team, RIKEN Center for Advanced Intelligence Project, Tokyo, Japan; 3 Data and Signal Processing Research Group, Department of Engineering, University of Vic - Central University of Catalonia, Barcelona, Spain; 4 School of Automation, Guangdong University of Technology, Guangzhou, China; 5 Faculty of Medicine, Juntendo University, Tokyo, Japan; 6 Juntendo University Nerima Hospital, Tokyo, Japan; 7 Graduate School of Engineering, Saitama Institute of Technology, Fukaya, Japan; 8 Department of Psychiatry, University of Cambridge, Cambridge, United Kingdom; 9 College of Artificial Intelligence, Nankai University, Tianjin, China

**Keywords:** SOZ, iEEG, PU learning, Supervised learning

## Abstract

Epilepsy is a chronic disorder caused by excessive electrical discharges. Currently, clinical experts identify the seizure onset zone (SOZ) channel through visual judgment based on long-time intracranial electroencephalogram (iEEG), which is a very time-consuming, difficult and experience-based task. Therefore, there is a need for high-accuracy diagnostic aids to reduce the workload of clinical experts. In this article, we propose a method in which, the iEEG is split into the 20-s segment and for each patient, we ask clinical experts to label a part of the data, which is used to train a model and classify the remaining iEEG data. In recent years, machine learning methods have been successfully applied to solve some medical problems. Filtering, entropy and short-time Fourier transform (STFT) are used for extracting features. We compare them to wavelet transform (WT), empirical mode decomposition (EMD) and other traditional methods with the aim of obtaining the best possible discriminating features. Finally, we look for their medical interpretation, which is important for clinical experts. We achieve high-performance results for SOZ and non-SOZ data classification by using the labeled iEEG data and support vector machine (SVM), fully connected neural network (FCNN) and convolutional neural network (CNN) as classification models. In addition, we introduce the positive unlabeled (PU) learning to further reduce the workload of clinical experts. By using PU learning, we can learn a binary classifier with a small amount of labeled data and a large amount of unlabeled data. This can greatly reduce the amount and difficulty of annotation work by clinical experts. All together, we show that using 105 minutes of labeled data we achieve a classification result of 91.46% on average for multiple patients.

## Introduction

Epilepsy is a chronic neurological disorder of the brain that affects people globally. According to the Epilepsy Foundation, approximately 65 million people worldwide have epilepsy. The International League Against Epilepsy (ILAE) (Fisher et al. 2014) define epilepsy by any of the following conditions:

- At least two unprovoked (or reflex) seizures occurring > 24 h apart;
- One unprovoked (or reflex) seizure and a probability of further seizures similar to the general recurrence risk (at least 60%) after two unprovoked seizures, occurring over the next 10 years;
- Diagnosis of an epilepsy syndrome.

According to the definition given by the ILAE (Fisher et al. 2017), there are three types of epileptic seizures: focus onset, generalized onset, and unknown onset. In this article we target at focus onset, which consist of recurrent seizures together with abnormal discharge in focus brain areas. The patients suffering from focus seizures can be treated with daily medication to control the epileptic seizure frequency, but approximately 30% of focus seizure patients are refractory to medication (Sinha et al. 2016), although there are currently some measures to detect and intervene in epileptic seizures (Çetin 2020; Hejazi and Motie Nasrabadi 2019), but this is a kind of interventional measure, not an effective treatment. Hence removing the SOZ channel by surgery is considered as a common treatment. Before the surgery, the SOZ channel needs to be identified and it is also the most important factor affecting the outcome of the surgery.

To identifying the SOZ channel, physical exam, iEEG, magnetoencephalogram (MEG), functional magnetic resonance imaging (fMRI), combination modalities (Ebrahimzadeh et al. 2021) (e.g. EEG-fMRI), and other modalities are usually performed (Duncan et al. 2016). Considering the cost of MEG and the recording condition (the subject can not move freely), MEG is not practical for experimental work. Because the time resolution of fMRI is very low, the brain activities can not be completely recorded and this is a drawback for identifying the SOZ channel. The iEEG is recorded directly from the cortex, allowing clinical experts to effectively analyze brain activity. These advantages make iEEG a fundamental tool in the detection, diagnosis, and treatment of epilepsy.

In current clinical practice, for the task of identifying the SOZ channel, clinical experts still rely on the visual assessment of iEEG data for judgment and manual annotation, which is a time-consuming, difficult and subjective process. Therefore, for some hard data, the diagnostic results from different clinical experts are often not identical, and clinical experts need to spend much more time discussing and even voting on the diagnosis. In addition, the number of clinical experts is far from enough compared to the large number of patients (e.g. only about 700 clinical experts in Japan can do the diagnosis of epilepsy [The Japan Epilepsy Society]). Therefore, in the diagnosis of epilepsy, there is a very strong demand for high-accuracy diagnostic aids to alleviate these problems.

According to the iEEG collection period, there are two types of brain signals: (i) the interictal signal, which is recorded between epileptic seizures, and (ii) the ictal signal, which is recorded during an epileptic seizure. In this article, we only use the interictal brain signal data. The channel recorded from the SOZ is called SOZ channel, and the channel recorded from the non-SOZ is called non-SOZ channel. During the interictal periods, epileptic spikes are often used for the diagnosis of epilepsy, which can allow the classification of the SOZ and non-SOZ channel data. The International Federation of Societies for Electroencephalography and Clinical Neurophysiology (IFSECN) (Noachtar et al. 1999) give the following definitions of spikes:

- Sharp wave: A transient, clearly distinguished from background activity, with pointed peak at a conventional paper speed or time scale and duration of 70 ± 200 ms, i.e. over 1/4 ± 1/5 s approximately.
- Spike: A transient, clearly distinguished from background activity, with pointed peak at a conventional paper speed or time scale and a duration from 20 to under 70 ms, i.e. 1/50 ± 1/15 s, approximately.
- Slow wave: Wave with duration longer than alpha waves, i.e. over 1/8 s.
- Spike-and-slow-wave complex: A pattern consisting of a spike followed by a slow wave.
- Multiple spike complex: A sequence of two or more spikes.
- Polyspikes-and-slow-wave complex: A sequence of two or more spikes associated with one or more slow waves.

In recent years, a variety different kinds of methods are proposed for SOZ identification, some traditional methods such as template matching (Lodder and van Putten 2014; Jing et al. 2016; Grouiller et al. 2011; Jin et al. 2014), dictionary learning (Spyrou and Sanei 2016), functional brain connectivity (Yu et al. 2020), classification (Sharma et al. 2015; Johansen et al. 2016) and other methods. With the rapid development of machine learning, classification methods based on neural networks show an expected performance in the SOZ identification problem. Usually, the first step in designing a classification system is feature extraction. For that, Discrete wavelet transform (DWT) (Das and Bhuiyan 2016; Deivasigamani et al. 2016; Chen et al. 2015; Zhou et al. 2012), Entropy (Sharma et al. 2015; Bao et al. 2009), Fourier transform (FT) (Singh and Pachori 2017), EMD (Itakura and Tanaka 2017; Gupta and Pachori 2019), Bispectrum (Acharya et al. 2019), Lempel-Ziv (Borowska 2021) or Filters (Itakura et al. 2018) are often used. The resolution in time and frequency is the main advantage of DWT, which makes it especially suitable for the analysis of the non-stationary signals (e.g. iEEG). EMD method decomposes a signal into intrinsic mode functions (IMFs), which are the modulated components of amplitude and frequency. Epilepsy is caused by excessive electrical discharges in brain cells, resulting in changes in the synchronization of the electroencephalography (EEG). Because entropy measures the degree of complexity of a signal, it can be used to extract features of epilepsy brain signals. The next step to design a classification system is to model the signals characterized by their features. This is called the classification step, in which K-nearest neighbor (KNN), probabilistic neural network (PNN), SVM, FCNN, CNN, and other methods are usually used.

In addition, based on the neural networks, there are also some end-to-end methods, it can train a complex model to integrate the features extraction and the classification processes (Daoud and Bayoumi 2019). Considering that iEEG is one-dimensional data, the method (Li et al. 2019) used the one-dimensional CNN model to perform the feature extraction and classification functions at the same time. Method (Fraiwan and Alkhodari 2020) uses the long short-term memory (LSTM) model to extract features of time series in iEEG data and perform the classification task. Moreover, the ensemble learning method (Jukic et al. 2020) is also used in the task of SOZ identification. Compared with classification models with feature extraction, the end-to-end model can avoid the manual design of feature extraction algorithms.

In our study, it is mainly derived from real clinical practice, the rationality and medical interpretation of the method need to be considered. Even the manual feature extraction process can be avoided by using end-to-end machine learning methods, but the entire operation is similar to a black box, and the specific processing method in the middle is not clear. The goal of this study is to localize the seizure onset zone (SOZ), in medicine, due to the SOZ containing more abnormal discharges, entropy is used as the feature extraction method. SOZ and Non-SOZ have some differences in the frequency domain, which prompted us to use the STFT as another feature extraction method. In the computer field, there are indeed many different feature extraction methods, but under the premise of considering the corresponding medical interpretation, the above methods are chosen for feature extraction. In our experiments, several typical supervised learning methods (SVM, FCNN, and CNN) are used for classification, but there are still some problems. Supervised learning is highly dependent on high-quality data with labels, but in the brain signal field, data annotation is a very time-consuming, difficult, and experience-based task. In clinical practice, for some hard data, the annotation results given by different clinical experts are often not identical. Hence, we introduce the PU learning method to further reduce the workload of clinical experts (Zhao et al. 2018). By using PU learning, we can learn a binary classifier with a small amount of labeled data and a large amount of unlabeled data to reduce the amount of data annotation required. For those hard data, we can treat it as unlabeled data, thus avoiding further the discussion and voting process.

The rest of the article is organized as follows: Section II describes the methods used, including the feature extraction and classification steps. In Section III we present the two datasets used in this article. Section IV contains the experimental results, Section V presents a discussion about the methods, while Section VI along with the conclusions.

## Methods

**Fig. 1:**
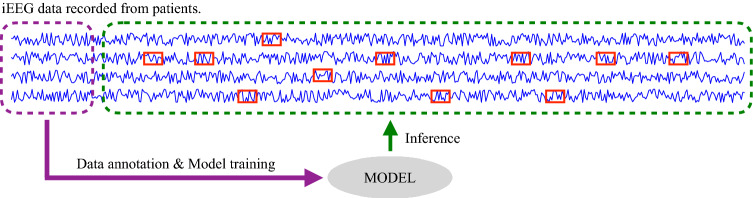
Method: On the left, a small block of annotated iEEG data (purple dotted line box) labeled by an expert. On the right, the prediction on the rest of the data (green dotted line box), which include the channel and period information. The red line box means that this part of the iEEG is classified as SOZ data, according to the model derived from the annotated data

The brain disorders are usually diagnosed by visual inspection of EEG/iEEG. For epilepsy, EEG is used to diagnose and iEEG is used for SOZ identification. EEG/iEEG analysis for assisting in the diagnosis of epilepsy has been used since about 30 years ago. One of the first works is Gotman (1990) and since then, other works have been published (Oikonomou et al. 2007; Exarchos et al. 2006; Wilson and Emerson 2002; Gotman 1999). Regardless of the EEG and iEEG diagnostic process, clinical experts need long-term visual inspection to analyze the signals. In this article, we propose to use some data labeled by experts to train a model that will be used to label the remaining data. The method is depicted in Fig. 1.

### Feature extraction

Many methods have been proposed to extract epileptic features from the iEEG. In the feature extraction step, we are interested not only in obtaining the most discriminating features, but also in the medical interpretation of them, as this is a very important aspect for clinical experts. In this article, we propose a method of feature extraction based on the short-time Fourier transform (STFT), and we also introduce another method originally proposed in Akter et al. (2020), which calculates several entropy versions in different frequency bands.

#### Feature extraction by using filters and entropy

The features of the raw iEEG are extracted through the use of seven different band-pass filters and eight different entropies, corresponding to different brain states. The band-pass filters, which cover the frequency bands commonly used is Delta: 0.5 to 4 Hz, Theta: 4 to 8 Hz, Alpha: 8 to 13 Hz, Beta: 13 to 30 Hz, Gamma: 30 to 80 Hz, Ripple: 80 to 250 Hz, Fast Ripple: 250 to 600 Hz. Different from scalp EEG, in iEEG, the high frequency components (HFO) also play important role in identifying SOZ, thus we use the above seven frequency bands (Liu et al. 2018; Brázdil et al. 2017; Hao et al. 2021). One of the datasets, the Bern-Barcelona, is already processed and band-pass filtered between 0.5 and 150 Hz. Therefore, in that case, the Fast Ripple band is not present and the Ripple band covers the frequencies from 80 to 150 Hz, instead of 80 to 250 Hz.

**Fig. 2:**
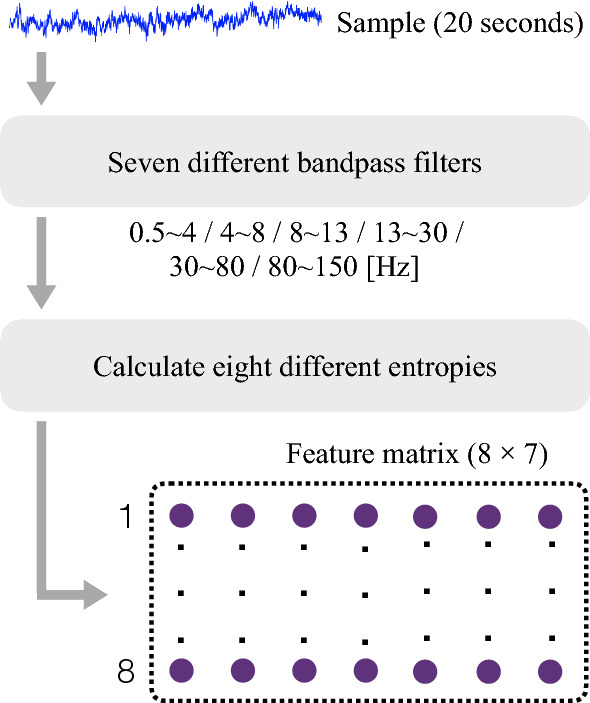
Feature extraction procedure, using filtering and entropies calculation (example for the Juntendo dataset)

To extract the features from the iEEG, we first split it in segments of 20 s, called samples. Then each sample is filtered using the seven band-pass filters (six for Bern-Barcelona dataset). Finally, eight different entropies are calculated for each of the frecuency bands, obtaining a feature matrix containing 8 $\times$ 7 features (8 $\times$ 6 for Bern-Barcelona dataset). The complete flow chart of feature extraction is shown in Fig. 2.

The eight entropies (Itakura et al. 2018) calculated are the following: Shannon entropy, Renee entropy, Generalized entropy, Phase entropy (two types), Approximate entropy, Sample entropy and Permutation entropy.

#### Feature extraction by using short time Fourier transform

**Fig. 3:**
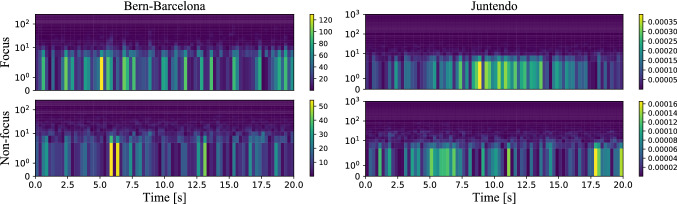
Examples of STFT. On the left, Bern-Barcelona data. On the right, Juntendo data. Window length is one second, with 80% overlap. For each subfigure, the X-axis is 0-20 s and the Y-axis (in log scale) is 0-256 Hz for Bern-Barcelona data and 0-1,000 Hz for Juntendo data

Time frequency analysis is a method to analyze signals from another perspective. In clinical practice, epileptic spikes are used to diagnose epilepsy, allowing to classify the SOZ. However, due to the noise in the iEEG, spikes are hard to identify in time domain, and frequency domain is used to ease that task. The non-stationarity of iEEG does not allow us to use ordinary time frequency analysis directly. In the article, we use STFT as a method for feature extraction in frequency domain. STFT divide the iEEG signal into short segments and then computes the Fourier transform on each one. By using STFT method, we can obtain the frequency information in a short period of time. One example of the STFT samples of Bern-Barcelona and Juntendo dataset is shown in Fig. 3

### Classification

In the article, we use three typical supervised learning methods for classification: SVM, FCNN and CNN. By using labeled iEEG data that annotated by clinical experts, we can train a classification model and then, by using it, we can predict the label of the new iEEG data. In addition, to solve the problem of labelling iEEG data, we also introduce the PU learning method for the SOZ identification problem.

#### Supervised learning

The principal characteristics and parameters of the supervised learning methods are presented succinctly here. The SVM method (Cortes and Vapnik 1995) maps the input data to a high dimensional features space, and then finds the hyper-plane in the features space allowing to separate the examples of each class.

The second classifier used in this article is FCNN model, the parameters are as follows: the size of the input layer is 48/56 (Bern-Barcelona/Juntendo), the size of the hidden layer one is 32, the size of the hidden layer two is 32, the size of the output layer is 2, the loss function is the binary cross entropy, the optimizer is the Adaptive Moment Estimation (Adam), with a batch size of 64.

Finally, the third classifier is CNN model, with the following parameters: the size of the input image is 8$\times$6/8$\times$7 (Bern-Barcelona/Juntendo), the size of the convolution kernel is 3$\times$3, the number of convolutional filters is 32, the size of the max pooling is 2$\times$2, the size of the hidden layer is 128, the size of the output layer is 2, the loss function is the categorical cross entropy, the optimizer is Adam, with a batch size of 64.

#### PU learning

With a large amount of labeled data, the traditional supervised learning methods show a high performance in classification results. However, in the medical field, data labels are assigned by clinical experts through visual inspection. This is a complicated process and often we need multiple clinical experts to give their opinion at the same time and vote to determine the final outcome of the label. To solve this problem and reduce the workload of clinical experts, several options can be considered. One option is to apply some data augmentation method on the labeled data to generate more artificial data with their corresponding label (Dinarès-Ferran et al. 2018; Zhang et al. 2019). This method needs, however, to have already labeled examples of both classes. To avoid this, we introduced the PU learning method for the problem of SOZ classification only using only a small set of labeled SOZ data.

**Fig. 4:**
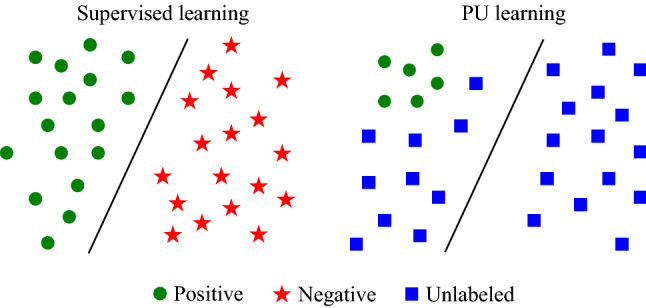
Model of supervised learning and PU learning

In Fig. 4 we represent the traditional model of supervised learning (left), in which we have two types of data (in a two-class scenario) and each data has its own label. On the other hand, for the PU learning model (right), we have two type of data (again, in a two-class scenario): a small amount of labeled data (positive class labels), while the rest is unlabeled data (from unknown class). This is the definition of PU learning (Liu et al. 2002):

Given a set of examples of a particular class P (named the positive class) and a set of unlabeled examples U, which contains both class P and non-class P (called the negative class) instances, the goal is to build a binary classifier to classify the test set T into two classes, positive and negative. There are some similarities between the PU learning and semi-supervised learning, which is also used in medical field (Shah et al. 2018), but unlike to the semi-supervised learning, PU learning only needs to give label to one kind of data.

Depending on how the problem of an unlabeled data type is solved, the learning process of PU can be divided into two groups. The first one is based on finding reliable negative data (RN) from unlabeled data. Then, by using the positive data and the RN data, the PU learning can be considered as a binary classifier (Blum and Mitchell 1998; Liu et al. 2002; Li and Liu 2003). The second one considers the unlabeled data as negative data, and the weight of the negative data is adjusted in the loss function (Liu et al. 2003; Elkan and Noto 2008; Lee and Liu 2003). This is the method used in the experiments. Description and mathematical details are as follows:

**Problem settings:** Let $\mathbf{x}$ be the input vector, which is calculate by filtering and calculating the entropies on the STFT from a 20-s iEEG data, and $y \in \{ \pm 1 \}$ be the class label. In the experiments, $+1$ stands for SOZ data and $-1$ stands for non-SOZ data. The class conditional distributions of SOZ and non-SOZ data are denoted by $p_{p}(\mathbf{x})$ and $p_{n}(\mathbf{x})$ respectively, defined by

1 $$\begin{aligned} \begin{aligned} p_{p}(\mathbf{x})&= p(\mathbf{x}\mid y = +1),\\ p_{n}(\mathbf{x})&= p(\mathbf{x}\mid y = -1). \end{aligned} \end{aligned}$$

The prior probabilities for SOZ and non-SOZ data are denoted by $\pi _{p} = p(y = +1)$ and $\pi _{n} = p(y = -1)$ with $\pi _{n} = 1 - \pi _{p}$. In this work, $\pi _{p}$ is assumed to be known in advance. The marginal distribution of unlabeled data (i.e. the distribution of SOZ and non-SOZ data) is defined by

2 $$\begin{aligned} p(\mathbf{x}) = \pi _{p} p_{p}(\mathbf{x}) + \pi _{n} p_{n}(\mathbf{x}). \end{aligned}$$

**Risk estimators:** We use the empirical unbiased risk estimator, proposed by Du Plessis et al. (2014), Du Plessis et al. (2015), Kiryo et al. (2017). The $g(\cdot )$ denotes the binary classification function and $\ell (g(\mathbf{x}), \pm 1)$ is the loss function. Therefore, $\hat{R}_{p}^+ (g)$ and $\hat{R}_{n}^- (g)$ denote the empirical risks for SOZ and non-SOZ data, respectively. The empirical risk for SOZ data, i.e., $\hat{R}_{p}^+ (g)$, is calculated by (3). Accordingly, the empirical risk for non-SOZ data, i.e., $\hat{R}_{n}^- (g)$, is calculated by (4). Then, the risk estimator is defined by (5).

3 $$\begin{aligned}&\hat{R}_{p}^+ (g) = \mathbb {E}_{\mathbf{x}\backsim p_{p}(\mathbf{x})} \ell (g(\mathbf{x}), +1), \end{aligned}$$

4 $$\begin{aligned}&\hat{R}_{n}^- (g) = \mathbb {E}_{\mathbf{x}\backsim p_{n}(\mathbf{x})} \ell (g(\mathbf{x}), -1). \end{aligned}$$

5 $$\begin{aligned}&\hat{R}_{pn}(g) = \pi _{p} \hat{R}_{p}^+ (g) + \pi _{n} \hat{R}_{n}^- (g), \end{aligned}$$

Because we only have the label of SOZ data, the distribution of non-SOZ data is unknown. Therefore, $\hat{R}_{n}^- (g)$ can not be computed straightforwardly in the equation (4). However, by using the equation (2), the distribution of non-SOZ data can be represented by

6 $$\begin{aligned} \pi _{n}p_{n}(\mathbf{x}) = p(\mathbf{x}) - \pi _{p}p_{p}(\mathbf{x}). \end{aligned}$$

Hence, the empirical risk for non-SOZ data can be computed by

7 $$\begin{aligned} \pi _{n} \hat{R}_{n}^-(g) = \hat{R}_{u}^- (g) - \pi _{p} \hat{R}_{p}^- (g), \end{aligned}$$

where $\hat{R}_{u}^- (g)$ and $\hat{R}_{p}^- (g)$ are the empirical risks under the distribution of unlabeled data and SOZ data, respectively, defined by

8 $$\begin{aligned} \begin{aligned} \hat{R}_{u}^- (g)&= \mathbb {E}_{\mathbf{x}\backsim p(\mathbf{x})} \ell (g(\mathbf{x}), -1), \\ \hat{R}_{p}^- (g)&= \mathbb {E}_{\mathbf{x}\backsim p_{p}(\mathbf{x})} \ell (g(\mathbf{x}), -1). \end{aligned} \end{aligned}$$

Finally, the risk estimator in equation (5) can be indirectly approximated by

9 $$\begin{aligned} \hat{R}_{pu}(g) = \pi _{p} \hat{R}_{p}^+ (g) + \hat{R}_{u}^- (g) - \pi _{p} \hat{R}_{p}^- (g). \end{aligned}$$

In general, $g(\mathbf{x})$ can be any classifier function, such as linear discriminant analysis (LDA), SVM, FCNN or others. Due to the recent great success of neural networks, in this work we use a three-layer FCNN as the binary classifier function $g(\mathbf{x})$. Based on the objective function presented in equation (9), we can thus easily employ the BP (backpropagation) algorithm to learn the deep neural network for the PU problem.

## Dataset

Two datasets are used to evaluate our methods. One is the public dataset of Bern-Barcelona (Andrzejak et al. 2012), which is recorded from Department of Neurology, University of Bern, Switzerland. Retrospective EEG data analysis has been approved by the ethics committee of the Kanton of Bern. To our knowledge, this is the only publicly available dataset for classifying iEEG into SOZ and non-SOZ channels in epilepsy. The other dataset is recorded from Juntendo University Hospital (Tokyo, Japan), which is same as the article (Akter et al. 2020). The dataset is recorded under the approval of the Juntendo University Hospital Ethics Committee and the Tokyo University of Agriculture and Technology Ethics Committee.

### Bern-Barcelona dataset

The dataset includes the iEEG recorded from five patients, who have long standing pharmacoresistant temporal lobe epilepsy and are candidates for epilepsy surgery. iEEG is recorded using AD-TECH (Racine, WI, USA) device with a sampling rate of 1,024 Hz, and down-sampled to 512 Hz prior to further analysis. All data are re-referenced against the median of all the channels and permanent artifacts are confirmed by visual inspection. No subject or channel position information is provided for this data set, and the 20-s segments come from any time position. Thus, the data is a mixture of them. The channels defined as the SOZ channels, where the first changes of the iEEG signal were detected, were visually inspected by at least two clinical experts. The other channels are defined as non-SOZ channels.

**Fig. 5:**
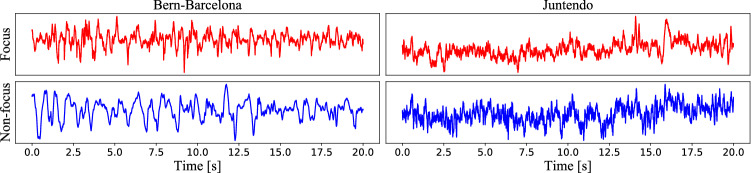
SOZ and non-SOZ samples, the sampling rate of Bern-Barcelona is 512 Hz and the sampling rate of Juntendo is 2,000 Hz

The initial dataset contains 7,500 SOZ and 7,500 non-SOZ samples, with each sample lasting 20 s, and filtered by a fourth-order Butterworth filter between 0.5 and 150 Hz. In order to minimize the phase distortions, forward and backward filtering is used. An example of SOZ and non-SOZ samples is shown in Fig. 5, left panels.

### Juntendo dataset

This dataset includes four patients who are suffering from epilepsy caused by focus cortical dysplasia. The demographic information of each patient is shown in Table 1, including age, gender, lesion site and location. The dataset consists of the interictal iEEG recorded during two hours at a sampling rate of 2,000 Hz. The subdural electrodes (four-mm diameter and ten-mm distance) (UNIQUE MEDICAL Co, Tokyo, Japan) are used to implant and cover the surface over the adjacent cortex. The label (SOZ or non-SOZ) was assigned by clinical experts after visual inspection. For this dataset, we know the code of the subjects and the positions of the channels. Four patients (namely patients 1, 2, 3 and 4) with SOZ channel number of 3, 3, 6, and 10, and randomly select 3, 3, 6, and 10 non-SOZ channels, then split the iEEG data into 20-s segments, each patient has 2,160, 2,160, 4,320, and 7,200 samples (half of SOZ and non-SOZ). An example of SOZ and non-SOZ samples for the Juntendo dataset is shown in Fig. 5, right panels.

**Table 1:** Summary of demographic information of each patient

Patient	Age	Gender	Lesion site	Location
P1	5	F	Lt dorsal superior temporal gyrus	Cortical surface
P2	6	F	Rt dorsal middle frontal gyrus	Cortical surface
P3	5	F	Lt cingulate gyrus	Bottom of sulcus
P4	39	F	Lt dorsal superior frontal gyrus	Bottom of sulcus

## Experimental results

Bern-Barcelona and Juntendo dataset were used to evaluate our proposed method. A 10-fold cross-validation strategy was implemented for the Bern-Barcelona dataset because it contains patients, channels, and time mixed together, we only know the label of SOZ and non-SOZ, but not their location or patient identification. In PU learning experiments, we have 13,500 training data. Among this data, 2,142 SOZ samples (which represents the about 15.87% of the training data) are selected as labeled data (the rest is unlabeled data). In order to compare the performance of the PU learning, we performed an experiment of small training data, the model of FCNN (entropy) is used, the training data include 1,071 SOZ and 1,071 non-SOZ samples (same size to labeled data in PU learning) and the test data include 1,500 samples (same size to the test data in PU learning).

In Juntendo dataset, the number of channels is different for each patient, hence the available samples vary among them: 2,160 for patient 1; 2,160 for patient 2; 4,320 for patient 3 and 7,200 for patient 4. In all the cases, half of the samples are SOZ and the other half are non-SOZ. We use the first 105 minutes to build the training dataset (87.5% of the data), and the rest 15 minutes to build the test dataset (the total recorded time was 120 minutes). In the PU learning method, we randomly select 300, 300, 600, 1000 (for patients 1 to 4, respectively, representing again the 15.87% of the training data) labeled SOZ samples, and the rest of the data are considered unlabeled data. Same to the Bern-Barcelona, we performed the experiment of small training data to compare the performance of PU learning, the model of FCNN (entropy) is used, the training data include 300, 300, 600, and 1,000 samples (half of SOZ and non-SOZ) for patients 1 to 4, and the test data also used the last 15 minutes (same to the test data in PU learning).

**Table 2:** Classification results, accuracy [%] over last 10 epochs (Mean ± Standard deviation, best result in bold)

Model	Bern-Barcelona	Juntendo P1	Juntendo P2	Juntendo P3	Juntendo P4
SVM (Entropy)	80.81 ± 1.60	85.56	65.56	81.30	92.44
FCNN (Entropy)	80.06 ± 1.58	91.67 ± 0.93	78.04 ± 0.97	80.44 ± 2.10	90.52 ± 0.16
CNN (Entropy)	83.80 ± 0.11	93.89 ± 0.65	81.37 ± 0.97	**89.11** ± 0.91	**94.66** ± 0.29
CNN (STFT)	**88.14** ± 0.12	**96.74** ± 3.77	**91.52** ± 1.10	81.80 ± 0.82	93.76 ± 0.95
PU & FCNN (Entropy)	76.91 ± 0.22	87.02 ± 1.20	74.31 ± 4.10	79.00 ± 1.59	87.20 ± 0.22
Small training data	70.61 ± 0.18	71.70 ± 3.16	61.15 ± 1.68	73.11 ± 0.20	84.60 ± 0.18

**Fig. 6:**
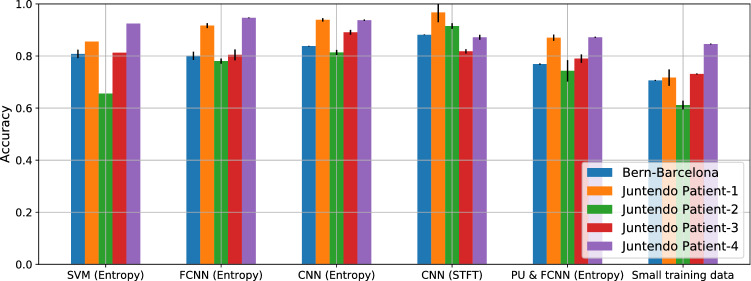
Illustrative experimental results (Bern-Barcelona and each patient in Juntendo)

The classification accuracy results obtained for the two datasets and each of the proposed models are shown in Table 2 and Fig. 6. Due to the Bern-Barcelona dataset is evaluated by 10-fold cross-validation, the results are averaged twice, the first is the average of 10-fold cross-validation, and the second is the last 10 epoch average.

## Discussion

In order to reduce the workload of the clinical expert, diagnostic aids of the SOZ classification are proposed, based on several feature extraction methods and classification models. In the feature extraction step, we use two different approaches: (i) various filtering and entropy calculations; and (ii) STFT. We are not only concerned by the effect of feature extraction, but also by ensuring its medical interpretation and making it accessible to clinical experts, which is a key point in clinical practice. The band-pass filters that we selected cover the physiological frequency bands commonly used in iEEG, which are therefore well known and accepted by clinical experts. This time we hope to mine as much information as possible from the original data, the features from the low-frequency to high-frequency are extracted and fed into the neural network model, allowing the model to find more effective information from the features. On the other hand, since epilepsy is caused by an abnormal discharge of brain cells, we use entropy as a characteristic because of its ability to measure the predictability of a signal, being also understandable for clinical experts. The reason for using STFT is based on the role of spikes in epilepsy. Because the identification of spikes in the time domain is very complicated, we propose to use time frequency analysis to do so. In the classification step, we compared several typical supervised learning methods. From the results, we can see that the more complex network model (CNN) shows better performance, almost regardless of the features extracted from the signals.

Supervised learning relies on high-quality labeled data, but the iEEG annotation is time-consuming, difficult and experience-based work. In particular, some data require multiple clinical experts to spend more time discussing and voting to make the final diagnosis. In order to further reduce the workload of clinical experts, we introduce the PU learning model to reduce the requirements for data annotation. By using the PU learning model, only part of SOZ data annotation is required. Compared with the commonly used supervised learning, the PU learning model can improve the work of data annotation. Table 3 is a comparison of supervised learning and PU learning methods, as shown in the table, PU learning can reduce the amount of annotation data, and due to the annotated sample being freely chosen, hard sample annotation also can be avoided. To investigate the effect of the PU strategy, we run another experiment in which we used FCNN (Entropy) as a classifier, training with the same amount of data used in the PU & FCNN (Entropy) case, but now using only labeled data and not unlabeled data (i.e., we didn’t use the PU strategy). This experiment is the same as FCNN (Entropy) in Table 2, but using a small dataset for training. To discuss the results, let’s focus on a single case, the Bern-Barcelona dataset. We can see in Table 2 that using all the available data we have the best result, as expected (80.06%). But we can also see that using PU strategy with only 15.9% of labeled data from SOZ class, we obtain a very good result (76.91%), with a small drop in accuracy of about 3.15%. Finally, using the same 15.9% of training data and testing in the same conditions as before (but now without using PU), the accuracy drops significantly to 70.61%, which is about 10% lower than the first case and 6% lower than using the PU strategy. Similar effect can be observed in all the subjects of the Juntento dataset. Therefore, thanks to PU we can train a classifier using a small amount of labeled data (SOZ signal) and a large amount of unlabeled data (SOZ and non-SOZ signal) keeping reasonable and useful classification results and reducing the workload of clinical experts.

**Table 3:** Comparison of method performance in the SOZ and non-SOZ iEEG data classification

Reference	Method	Annotation Ratio (%)	Avoids Hard Sample	Accuracy (%)
Acharya et al. (2019)	Bispectrum, LS-SVM	100		87.93
Borowska (2021)	Lempel-Ziv, LS-SVM	100		86
Gupta and Pachori (2019)	EMD, LS-SVM	100		83.12
Proposed	STFT, CNN	100		88.14
	Entropy, FCNN	100		80.06
	Entropy, FCNN & PU	15.87		76.91

Although the proposed methods exhibits expected performance, there are still some limitations. The proposed method cannot be used in a patient-independent setting which is important for real clinical data application. For each patient, we still need clinical experts to perform part of the data annotation. By using PU learning we can further reduce the annotation workload of clinical experts, but at the same time, it has some shortcomings. PU learning needs to know the proportion of the positive data in the unlabeled data and the error of this proportion value affects the classification performance. This will limit the practical application of the method. Due to the difficulty of data collection and annotation, in the experiment, we can only evaluate the proposed method with limited patient data. In this article, we propose methods to classify the SOZ and non-SOZ data and return the classification results to clinical experts as an auxiliary basis for diagnosis. But the classification result is an intermediate process for clinical experts to identify the SOZ, if most of the segment samples classified as SOZ are appear in some channels, those channels will have a greater probability of being identified as SOZ. In this way, the classification results can help the clinical experts to identify the SOZ channels.

## Conclusion

In this article, we propose methods to classify the SOZ and non-SOZ data. In the feature extraction, filtering with entropy calculation and STFT are used. In the classification, various models (SVM, FCNN and CNN) are used as the classifier and from the experimental results our method obtained the best result compared with other published works. In order to further reduce the annotation workload and difficulty of clinical experts, we introduce the PU learning for SOZ data classification, by using PU learning, only a small part of SOZ data is required to be annotated. For data that is difficult to diagnose, it can be processed as unlabeled data, thereby reducing the difficulty of data annotation. The proposed methods are evaluated using two datasets. As can be seen from the experimental results, the classification result obtained by the different methods is as expected. In the PU learning results, only 15.9% of labeled data from the SOZ class are used, the model obtain a very good result (76.91%). Compared to supervised learning, the result has a small drop in accuracy of about 3.15%. Compares to the supervise learning model with 15.9% of training data, the PU learning shows a better classification result. PU learning provides us with an opportunity to use a small amount of labeled data for SOZ and non-SOZ classification.

## References

[CR1] Fisher RS, Acevedo C, Arzimanoglou A, Bogacz A, Cross JH, Elger CE, Engel J, Forsgren L, French JA, Glynn M, Moshé SL, Perucca E, Scheffer IE, Tomson T, Watanabe M, Wiebe S (2014). ILAE official report: a practical clinical definition of epilepsy. Epilepsia.

[CR2] Fisher RS, Cross JH, D’souza C, French JA, Haut SR, Higurashi N, Hirsch E, Jansen FE, Lagae L, Moshé SL, Peltola J, Perez ER, Scheffer IE, Schulze-Bonhage A, Somerville E, Sperling M, Yacubian EM, Zuberi SM (2017). Instruction manual for the ILAE 2017 operational classification of seizure types. Epilepsia.

[CR3] Sinha N, Dauwels J, Kaiser M, Cash SS, Brandon Westover M, Wang Y, Taylor PN (2016). Predicting neurosurgical outcomes in focal epilepsy patients using computational modeling. Brain.

[CR4] Çetin M (2020). Model-based robust suppression of epileptic seizures without sensory measurements. Cognit Neurodynamics.

[CR5] Hejazi M, Motie Nasrabadi A (2019). Prediction of epilepsy seizure from multi-channel electroencephalogram by effective connectivity analysis using granger causality and directed transfer function methods. Cognit Neurodynamics.

[CR6] Ebrahimzadeh E, Shams M, Rahimpour Jounghani A, Fayaz F, Mirbagheri M, Hakimi N, Rajabion L, Soltanian-Zadeh H (2021). Localizing confined epileptic foci in patients with an unclear focus or presumed multifocality using a component-based EEG-fMRI method. Cognit Neurodynamics.

[CR7] Duncan JS, Winston GP, Koepp MJ, Ourselin S (2016). Brain imaging in the assessment for epilepsy surgery. Lancet Neurol.

[CR8] Noachtar S, Binnie C, Ebersole J, Mauguiere F, Sakamoto A, Westmoreland B (1999). A glossary of terms most commonly used by clinical electroencephalographers and proposal for the report form for the EEG findings, the international federation of clinical neurophysiology. Electroencephalogr Clin Neurophysiol.

[CR9] Lodder SS, van Putten MJ (2014). A self-adapting system for the automated detection of inter-ictal epileptiform discharges. PloS one.

[CR10] Jing J, Dauwels J, Rakthanmanon T, Keogh E, Cash S, Westover M (2016). Rapid annotation of interictal epileptiform discharges via template matching under dynamic time warping. J Neurosci methods.

[CR11] Grouiller F, Thornton RC, Groening K, Spinelli L, Duncan JS, Schaller K, Siniatchkin M, Lemieux L, Seeck M, Michel CM, Serge V (2011). With or without spikes: localization of focal epileptic activity by simultaneous electroencephalography and functional magnetic resonance imaging. Brain.

[CR12] Grouiller F, Thornton RC, Groening K, Spinelli L, Duncan JS, Schaller K, Siniatchkin M, Lemieux L, Seeck M, Michel CM, Serge V (2011). With or without spikes: localization of focal epileptic activity by simultaneous electroencephalography and functional magnetic resonance imaging. Brain.

[CR13] Spyrou L, Sanei S (2016) Coupled dictionary learning for multimodal data: an application to concurrent intracranial and scalp EEG, In: Proceedings IEEE-ICASSP, Shanghai, China, p 2349–2353

[CR14] Yu H, Zhu L, Cai L, Wang J, Liu C, Shi N, Liu J (2020). Variation of functional brain connectivity in epileptic seizures: an EEG analysis with cross-frequency phase synchronization. Cognit Neurodynamics.

[CR15] Sharma R, Pachori RB, Acharya UR (2015). An integrated index for the identification of focal electroencephalogram signals using discrete wavelet transform and entropy measures. Entropy.

[CR16] Johansen AR, Jin J, Maszczyk T, Dauwels J, Cash SS, Westover MB (2016) Epileptiform spike detection via convolutional neural networks, In: Proceedings IEEE-ICASSP, Shanghai, China, p 754–758 10.1109/ICASSP.2016.7471776 PMC5842703 29527131

[CR17] Das AB, Bhuiyan MIH (2016). Discrimination and classification of focal and non-focal EEG signals using entropy-based features in the EMD-DWT domain. Biomed Signal Process Control.

[CR18] Deivasigamani S, Senthilpari C, Yong WH (2016). Classification of focal and nonfocal EEG signals using ANFIS classifier for epilepsy detection. Int J Imaging Syst Technol.

[CR19] Chen D, Wan S, Bao FS (2015) Epileptic focus localization using EEG based on discrete wavelet transform through full-level decomposition, In: Proceedings MLSP, Boston, Massachusetts, USA, p 1–6

[CR20] Zhou J, Schalkoff RJ, Dean BC, Halford JJ (2012) Morphology-based wavelet features and multiple mother wavelet strategy for spike classification in EEG signals, In: Proceedings IEEE-EMBC, San Diego, California, USA, p 3959–3962 10.1109/EMBC.2012.6346833 23366794

[CR21] Sharma R, Pachori RB, Acharya UR (2015). Application of entropy measures on intrinsic mode functions for the automated identification of focal electroencephalogram signals. Entropy.

[CR22] Bao FS, Gao J-M, Hu J, Lie DY, Zhang Y, Oommen K (2009) Automated epilepsy diagnosis using interictal scalp EEG, In: Proceedings IEEE-EMBC, Minneapolis, Minnesota, USA, p 6603–6607 10.1109/IEMBS.2009.5332550 19963676

[CR23] Singh P, Pachori RB (2017). Classification of focal and nonfocal EEG signals using features derived from fourier-based rhythms. J Mech Med Biol.

[CR24] Itakura T, Tanaka T (2017) Epileptic focus localization based on bivariate empirical mode decomposition and entropy, In: Proceedings IEEE-APSIPA ASC, Aloft Kuala Lumpur Sentral, Malaysia, p 1426–1429

[CR25] Gupta V and Pachori RB (2019) A new method for classification of focal and non-focal EEG signals, In: Machine intelligence and signal analysis, Springer, Singapore, p 235-246

[CR26] Acharya UR, Hagiwara Y, Deshpande SN, Suren S, Koh JEW, Oh SL, Arunkumar N, Ciaccio EJ, Lim CM (2019). characterization of focal EEG signals: a review. Future Gener Comput Syst.

[CR27] Borowska M (2021). multiscale permutation lempel-ziv complexity measure for biomedical signal analysis: Interpretation and application to focal EEG signals. Entropy.

[CR28] Itakura T, Ito S, Tanaka T, Sugano H (2018). Effective frequency bands and features for epileptic focus detectionfrom interical electrocorticogram. IEICE Tech Rep IEICE Tech Rep.

[CR29] Daoud H, Bayoumi M (2019). Deep learning approach for epileptic focus localization. IEEE Trans Biomed Circuits Syst.

[CR30] Li B, Zhao X, Zhao Q, Tanaka T, Cao J (2019) A one–dimensional convolutional neural network model for automated localization of epileptic foci, In: Asia–Pacific signal and information processing association annual summit and conference (APSIPA ASC), p 741–744

[CR31] Fraiwan L, Alkhodari M (2020). Classification of focal and non-focal epileptic patients using single channel EEG and long short-term memory learning system. IEEE Access.

[CR32] Jukic S, Saracevic M, Subasi A, Kevric J (2020). Comparison of ensemble machine learning methods for automated classification of focal and non-focal epileptic EEG signals. Mathematics.

[CR33] Zhao X, Tanaka T, Wanzeng K, Qibin Z, Jianting C, Hidenori S, Noburu Y (2018) Epileptic focus localization based on iEEG by using positive unlabeled (PU) learning, In: Proceedings IEEE-APSIPA ASC, Honolulu, Hawaii, USA, p 493–497

[CR34] Gotman J (1990). Automatic seizure detection: improvements and evaluation. Electroencephalogr clin Neurophysiol.

[CR35] Oikonomou VP, Tzallas AT, Fotiadis DI (2007). A kalman filter based methodology for EEG spike enhancement. Comput Methods Programs Biomed.

[CR36] Exarchos TP, Tzallas AT, Fotiadis DI, Konitsiotis S, Giannopoulos S (2006). EEG transient event detection and classification using association rules. IEEE Trans Inf Technol Biomed.

[CR37] Wilson SB, Emerson R (2002). Spike detection: a review and comparison of algorithms. Clin Neurophysiol.

[CR38] Gotman J (1999). Automatic detection of seizures and spikes. J Clin Neurophysiol.

[CR39] Akter MS, Islam MR, Iimura Y, Sugano H, Fukumori K, Wang D, Tanaka T, Cichocki A (2020). Multiband entropy-based feature-extraction method for automatic identification of epileptic focus based on high-frequency components in interictal iEEG. Scientific Rep.

[CR40] Liu S, Gurses C, Sha Z, Quach MM, Sencer A, Bebek N, Curry DJ, Prabhu S, Tummala S, Henry TR, Ince NF (2018). Stereotyped high-frequency oscillations discriminate seizure onset zones and critical functional cortex in focal epilepsy. Brain.

[CR41] Brázdil M, Pail M, Halámek J, Plešinger F, Cimbálník J, Roman R, Klimeš P, Daniel P, Chrastina J, Brichtová E (2017). Very high-frequency oscillations: novel biomarkers of the epileptogenic zone. Ann Neurol.

[CR42] Hao J, Cui Y, Niu B, Yu L, Lin Y, Xia Y, Yao D, Guo D (2021). Roles of very fast ripple (500–1000 hz) in the hippocampal network during status epilepticus. Int J Neural Syst.

[CR43] Cortes C, Vapnik V (1995). Support-vector networks.. Mach Learn.

[CR44] Dinarès-Ferran J, Ortner R, Guger C, & Solé-Casals J (2018) A new method to generate artificial frames using the empirical mode decomposition for an EEG-based motor imagery BCI, Fronti Neurosci, 12, 308. 10.3389/fnins.2018.00308 PMC5958196 29867320

[CR45] Zhang Z, Duan F, Solé-Casals J, Dinarès-Ferran J, Cichocki A, Yang Z, Sun Z (2019). A novel deep learning approach with data augmentation to classify motor imagery signals. IEEE Access.

[CR46] Liu B, Lee WS, Yu PS, Li X (2002) Partially supervised classification of text documents, In: Proceedings ICML, Sydney, Australia, p 387–394

[CR47] Shah MP, Merchant S, Awate SP (2018) Abnormality detection using deep neural networks with robust quasi-norm autoencoding and semi-supervised learning, In: Proceedings IEEE-ISBI, Washington, D.C., USA, , p 568–572

[CR48] Blum A, Mitchell T (1998) Combining labeled and unlabeled data with co-training, In: Proceedings COLT, Madison, Wisconsin, USA, p 92–100

[CR49] Li X, Liu B (2003) Learning to classify texts using positive and unlabeled data. In: Proceedings IJCAI, Acapulco, Mexico, p 587–592

[CR50] Liu B, Dai Y, Li X, Lee WS, Yu PS (2003) Building text classifiers using positive and unlabeled examples, In: Proceedings IEEE-ICDM, Melbourne, Florida, USA, p 179–186

[CR51] Elkan C, Noto K (2008) Learning classifiers from only positive and unlabeled data, In: Proceedings ACM-SIGKDD, New York, NY, USA, p 213–220

[CR52] Lee WS, Liu B (2003) Learning with positive and unlabeled examples using weighted logistic regression, In: Proceedings ICML, vol 3, Washington D.C., USA, p 448–455

[CR53] Du Plessis MC, Niu G, Sugiyama M (2014) Analysis of learning from positive and unlabeled data, In: Proceedings NIPS, Montréal, Canada, p 703–711

[CR54] Du Plessis M, Niu G, Sugiyama M (2015) Convex formulation for learning from positive and unlabeled data, In: Proceedings ICML, Lille, France, p 1386–1394

[CR55] Kiryo R, Niu G, du Plessis MC, Sugiyama M (2017) Positive-unlabeled learning with non-negative risk estimator, In: Proceedings NIPS, Long Beach, California, USA, p 1675–1685

[CR56] Andrzejak RG, Schindler K, Rummel C (2012). Nonrandomness, nonlinear dependence, and nonstationarity of electroencephalographic recordings from epilepsy patients. Phys Rev E.

